# Obstructive Sleep Apnea among Players in the National Football League: A Scoping Review

**DOI:** 10.4172/2167-0277.1000278

**Published:** 2017-11-23

**Authors:** April J Rogers, Kevin Xia, Kyaw Soe, Azizi Sexias, Felix Sogade, Barbara Hutchinson, Dorice Vieira, Samy I McFarlane, Girardin Jean-Louis

**Affiliations:** 1 Center for Healthful Behavior Change (CHBC), Division of Health and Behavior, Department of Population Health, New York University Medical Center, New York, NY 10016, USA; 2 Department of Health Service Administration, St. John’s University, Queens, NY 11439, USA; 3 Department of Medicine, Jersey Shore University Medical Center, Neptune, NJ 07753, USA; 4 Department of Cardiology, Health Service of Central Georgia, Macon, Georgia 31201, USA; 5 President of Chesapeake Cardiac Care, Annapolis, Maryland, 21401, USA; 6 Department of Medicine, Division of Endocrinology, State University of New York, Downstate Medical Center, Brooklyn, NY 11203, USA

**Keywords:** Obstructive sleep apnea, Sleep Apnea, Hypertension, Insufficient sleep

## Abstract

**Objective:**

Obstructive sleep apnea (OSA) is a common sleep-disordered breathing condition that has emerged as a significant public health problem given its increased prevalence over the past decade. The high prevalence of obesity and large waist circumference among NFL players are two risk factors that might contribute to the high susceptibility of football players to develop OSA. National Football League linemen might be particularly vulnerable since they tend to have a higher body mass index. In this scoping review, we aim to bring attention to the limited research regarding OSA among National Football League players and highlight the negative consequences of OSA in an attempt to increase awareness of the urgent need for further research in this area.

**Methods:**

Search terms associated with obstructive sleep apnea and football were used to examine Google Scholar, EMBASE, CINAHL, PubMed, ProQuest, and Web of Science Plus for relevant studies. All relevant studies were included and documented.

**Results:**

Findings included (n=4) studies of interest. All 4 studies revealed a near or slightly above 50% prevalence of OSA in the investigated cohorts (mostly retired NFL linemen). Most participants in the study (active NFL players) showed symptoms associated with a sleep-disorder breathing condition (snoring).

**Conclusion:**

OSA requires more attention from the research and medical community. As suggested by results in the 4 studies included in this paper, OSA and associated symptoms are prevalent in the NFL population. Further research is required to investigate the extent of OSA and OSA risk in this population. There is an urgent need to conduct OSA risk surveillance in the athletic community.

## Introduction

Sleep is vital for human survival and adequate bodily functioning [1,2]. When adequate sleep quality is experienced, the body responds in an efficacious manner as observed in optimal body metabolism and heightened cognitive awareness, which can promote better decisionmaking [3]. In the sports world, in order to be competitive and perform at optimal levels, it is paramount to receive efficient sleep [4]. In recent years, sleep-disordered breathing such as obstructive sleep apnea (OSA) has gained notoriety in the athletic community. OSA has been implicated as a medical condition that affects millions of Americans [5]. In the National Football League (NFL) community, the prevalence of OSA is estimated to range from 14% to 19%, and the prevalence in the general USA population is estimated at 2–5% [4,5]. During an apnea episode, which is characteristic among persons with OSA, the individual stops breathing for a short period of time resulting in forced awakening, disrupting the individual’s sleep and reduces total sleep time and compromises their overall sleep health [6]. The restriction of airflow leads to impairment of baroreceptor modulation of sympathetic nerve activity; such dysregulation can exacerbate arteriosclerosis, hypertension and other cardio-metabolic disorders [7].

OSA has been proposed as an important risk factor in the cause of death among NFL players such as Reggie White, who played professionally for the Philadelphia Eagles and the Green Bay Packers. Other notable NFL players diagnosed with OSA include Tony Dorsett, Warren Sapp, Aaron Taylor, Percy Harvin and Ja Marcus Russell [7]. Research has revealed that football players have a higher prevalence of obesity, de ined as body mass index (BMI) > 30 kg/m^2^ [8,9]. Obesity is among the many predictors of OSA risk [10]. Currently, there is limited information regarding the association of obstructive sleep apnea among American professional football players. Thus, the purpose of this scoping review is to evaluate the current literature on the impact of OSA among NFL players.

## Methods

**Figure 1:**
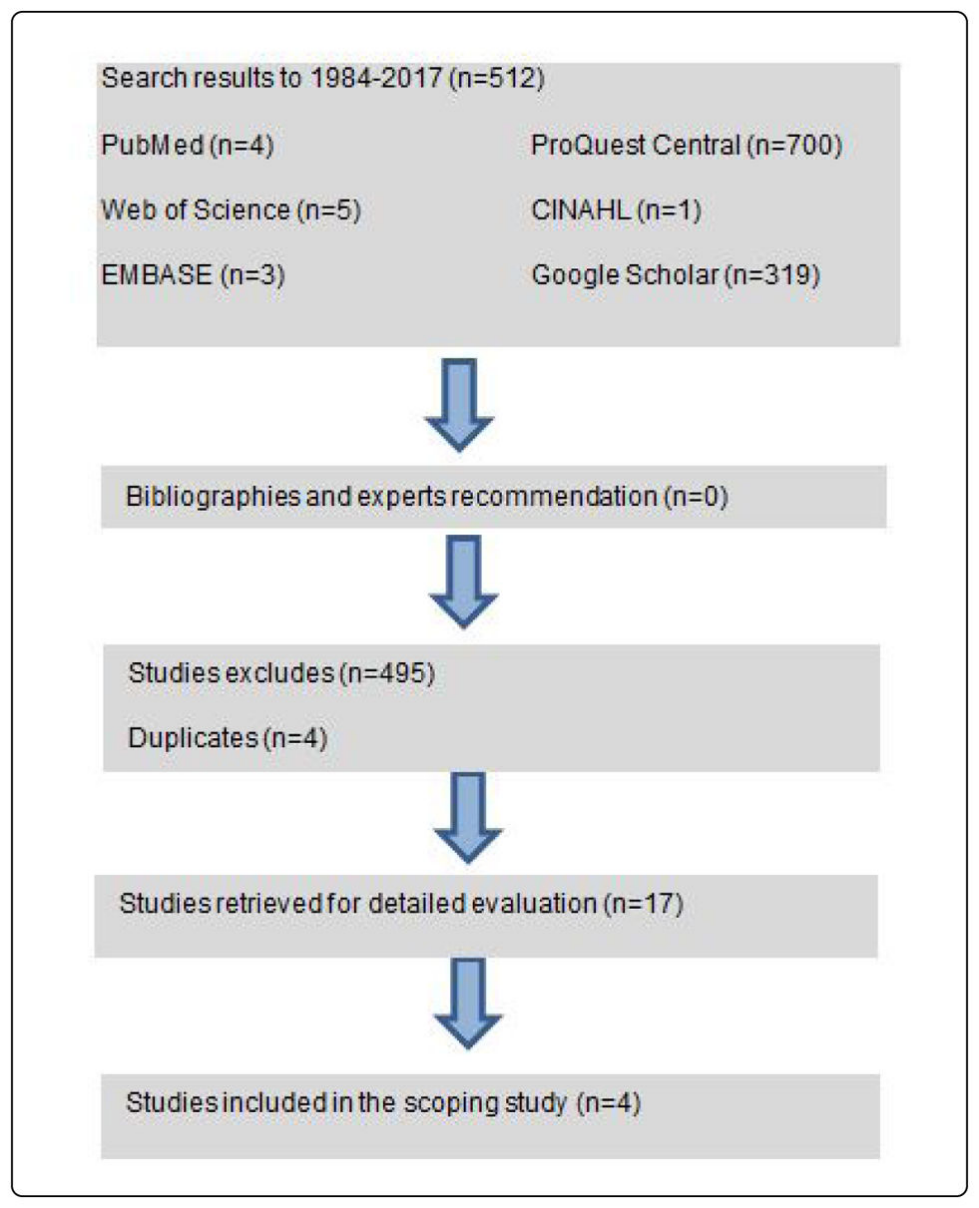
A flow chart using databases.

Research objectives were addressed using a scoping review methodology. The aim of a scoping review is to: 1) map relevant concepts and identify gaps in research [11] and 2) provide a comprehensive overview of the literature without engaging in the appraisal of multiple study outcomes [11]. The procedures of a scoping review consists of (1) identifying the research questions (2) identifying relevant studies, (3) study selection (4) charting the data (5) collating, summarizing and reporting the results and (6) an expert advisory consultation exercise [11]. Search terms were identified and agreed upon by authors and a medical librarian from New York University School of Medicine. Our literature search included a study of six databases; Google Scholar, EMBASE, CINAHL, PubMed, ProQuest, and Web of Science Plus. The following combination of terms was used to conduct a search in each database: (National football players or national football player OR professional football players or professional football player or national football league or NFL) and (Cardiovascular disease or cardiovascular diseases or heart disease or heart failure or vascular disease) and (Sleep disordered breathing OR apnea or apneas or sleep hypopneas) and (hypertension OR high blood pressure). A diagram illustrating the inclusion and exclusion of studies included is shown in Figure 1.

All articles were manually reviewed to identify any relevant studies. Grey literature was excluded; literature study was exclusive to peer-reviewed articles between 1984–2017. Citations were managed using bibliographic software manager endnote [11]. Eligibility criteria placed emphasis on whether studies provided a direct or broad description of the associations of obstructive sleep apnea among national league football players with cardiovascular disease and related sleep breathing disorders. The titles and abstracts of each citation were screened by the lead author; all relevant peer-reviewed articles were procured for full-text version.

## Results

Searches were carried out during the month of June 2017. All databases were searched independently, and all articles returned were compiled and saved in Endnote. All duplicates were removed, and after final screening and data extraction (n=4). Of the 512 references recorded, 495 were excluded during the initial screening process. The remaining 17 articles were reviewed in-depth and 4 articles were included in this scoping review. A flow chart of this process can be found (Figure 1).

Relevant information was extracted from each paper and charted to highlight the following: author, year of publication, study title, method, theme, outcome and limitations.

## Discussion

The purpose of this scoping study was to organize and evaluate the limited literature regarding obstructive sleep apnea among NFL players and to increase awareness of the adverse effects of untreated OSA. As noted from NFL recruitment surveys, average NFL linemen weigh over 300 lbs; and this is now the norm compared with 3 decades ago (300 players in 2017 over 300 lbs., 10 players in 1986) [12]. This trend starts at the college level and has continued on this trajectory. There is an unprecedented need for further studies on the health of NFL linemen, fueled by the unmeasured dangers of NFL athletes increasing in size, weight, and BMI. The widow of Reggie White has called for increased awareness of OSA through an education campaign launched by a foundation under his name as well as health initiatives promoted by different organizations in the league [13]. For clinicians, there is a paucity of peer-reviewed information on OSA among NFL players. Failure to treat OSA can increase the risk of stroke and cardiovascular disease [8]. The effects of OSA are rampant, not only regarding the quality of sleep and daytime performance, but there is also a tremendous influence on the circulatory system [8,14]. Repeated and recurrent nightly episodes of dyspnea from airflow collapse leads to retention of carbon dioxide and low oxygenation [15]. This can result in increased heart rate and respiratory drive provoked after many apneic cycles per hour per night of raised stress hormones and catecholamine that leads to elevated blood pressures [16]. Over time, the incremental effect would exacerbate comorbid factors such as preexisting hypertension, metabolic syndrome, dyslipidemia, coronary artery disease and the chances of mortality from a stroke, heart attack or both [16].

## Limitations

The data from the studies included in this scoping study were largely in support of the presence of OSA among linemen in the NFL. This is consistent with the hypothesis of the scoping study: NFL players are more predisposed to OSA than are non-NFL players. However, there are multiple confounding factors and limitations from the studies that should be further explored.

The power of the studies included is an important limitation. The study conducted by Rice et al. shows a negligible incidence of OSA among linemen and non-linemen. The study further revealed that investigators fell short on their recruitment goals. Drop out or inability to complete study for any number of reasons reduced the sample size even smaller.

Volunteer bias was an issue in several studies and was mentioned by Albuquerque et al. as a limitation in their study. Their study, which favored the hypothesis that NFL linemen suffered from a higher prevalence of OSA, could have been affected by the concerns of the participants that led them to volunteer for the study. The volunteers could have been concerned with having some or all of the symptoms of OSA or its associated comorbidities such as high blood pressure. The study, therefore, could have excluded those individuals that were not aware of their symptoms and or chose not to participate. Again, volunteer drop-out or inability to complete the study reduced the sample size.

Generalization of the collected data from the studies presently is difficult in part due to the limitations mentioned above, but may also be due to lack of specific factors such as ethnicity. Ethnicity of the participants was collected, but the percentage of each ethnic group regarding the presence of OSA was not reported. This may be because ethnicity was not seen as a risk factor in the development of OSA. There are even fewer studies done on whether certain races/ethnicities have a higher prevalence of developing OSA among NFL players. However, it is widely well documented that certain chronic comorbidities such as diabetes and hypertension do affect certain races/ethnicities greater, such as Blacks [8]. Other studies have found that minorities tend to experience less sleep quantity and quality than do whites [16]. Furthermore, since NFL participants were all male between the ages of 23–28 years, application of the findings to the general population must be done cautiously.

The accuracy and validity of using BMI as a predictor of obesity is questioned because it does not take into account muscle mass, bone density and body composition [15]. In addition to obesity, other attributes that put a linemen or non-linemen at risk for OSA could be dependent on other variables such as other comorbidities (diabetes and cardiovascular disease) [16]. Many studies used measurements such as neck circumference and hip-to-waist ratio in addition to BMI. Other researchers propose the use of DEXA scan for better estimation of body fat composition for determination of true obesity [15].

The accuracy and validity of home sleep apnea testing (HSAT) were called into question and this modality for OSA detection was commonly used across the studies in the scoping study. The gold standard for which HSAT is compared against is polysomnography in inpatient sleep study. In general practice, physicians would refer individuals suspected of OSA from the initial screening questionnaire to undergo overnight polysomnographic sleep study. This has been the gold standard to assess sleep apnea. In-patient sleep studies may not involve the physician directly over the course of the night but involves implementing multiple monitoring modalities. Some of the data recorded include rapid eye movement (REM) occurrence and brain wave activity via electroencephalogram (EEG), possible cardiac arrhythmias via electrocardiogram (ECG), carbon dioxide levels, muscle tone and whether substances have been utilized to induce sleep such as alcohol and medications. Compared with lab-based sleep studies, HSAT may be preferred because of convenience, accessibility, and lower costs (no overnight staff). Rice et al. state there was data loss (22 of 159) in their study from the single channel portable unattended home study reporting apnea/hypopnea episodes. The HSAT results in Rice et al. study were compared to results of the general population with OSA that was produced by polysomnography. Two different machines could have an unreported amount of variation. Furthermore, there are multiple types of HSAT and some studies reported which type was used while others did not.

## Conclusion

Lifestyle changes such as weight reduction to reduce OSA severity is widely known as a method to mitigate the symptoms of OSA. However, for most NFL linemen weight reduction may not be feasible for their active careers. For this population, greater emphasis needs to be placed on adherences to OSA treatment such as Continuous Positive Airway Pressure (CPAP). Noncompliance to CPAP should be closely followed by clinicians to investigate the root reasons if noncompliance occurs. If non-adherence to CPAP is in the form of discomfort or perceived stigma of wearing a mask, increased educational awareness and successful treatment testimonials can open opportunities to increase adherence. Further studies are required to improve the rate of screening NFL players for OSA risk. The potential impact of treating OSA may have a positive influence in athletic agility and increase players performance in addition to decreasing risk for cardiovascular disease and sudden cardiac death.

## Figures and Tables

**Table 1:** Collating, summarizing and reporting the result.

Authors	Year	Method	Themes	Outcome
Hyman et al.	2012	Logistic regression	Sensitivity and specificity of use of BMI for obesity	Of 129 NFL players, nearly half played linemen. After 1–32 years out from retirement most prevalent conditions were OSA and HTN; 42% reporting both
Albuquerque et al.	2010	Case control	Prevalence of SDB in retired NFL players	Of 257 retired NFL players; SDB was present in 52.3%. Defensive linemen were more likely to have SDB (61%vs. 46%; p= 0.02) than non-linemen
Rice et al.	2010	Cross sectional cohort study	Prevalence of SDB in active NFL players	Of 137 active NFL players recruited from 12 different teams 100% reported snoring; observed apneas in 23.9%; Moderate SDB in 4.4%; no significant differences between linemen and nonlinemen
Luyster et al.	2017	Retrospective analysis	Compared OSA risk in retired NFL players to CARDIA sleep study participants	Retired NFL players have greater prevalence of OSA

## References

[1] Ellenbogen JM (2005). Cognitive benefits of sleep and their loss due to sleep deprivation. Neurology.

[2] Valentino DJ, O’Donnell AE (2006). Reasons and referrals for sleep medicine consultation in obstructive sleep apnea. Chest.

[3] Williams NJ, Jean LG, Ravenell J, Seixas A, Islam N (2016). A community-oriented framework to increase screening and treatment of obstructive sleep apnea among blacks. Sleep Med.

[4] Ravenell J, Seixas A, Rosenthal DM, Williams O, Ogedegbe C (2016). Effect of birthplace on cardiometabolic risk among blacks in the Metabolic Syndrome Outcome Study (MetSO). Diabetol Metab Syndr.

[5] Diaz AM, Chatila W, Lammi MR, Swift I, D’Alonzo GE (2014). Determinants of CPAP Adherence in Hispanics with Obstructive Sleep Apnea. Sleep Disord.

[6] George CF, Kab V, Levy AM (2003). Increased prevalence of sleep-disordered breathing among professional football players. Solutions ss. Sleep apnea and NFL players. N Engl J Med.

[7] Luyster FS, Dunn RE, Lauderdale DS, Carnethon MR, Tucker AM (2017). Sleep-apnea risk and subclinical atherosclerosis in early-middle-aged retired National Football League players. Nat Sci Sleep.

[8] Buell JL, Calland D, Hanks F, Johnston B, Pester B (2008). Presence of metabolic syndrome in football linemen. J Athl Train.

[9] Rogers A, Ravenell J, Donat M, Sexias A, Ogedegbe C (2015). Predictors of obstructive sleep apnea risk among Blacks with metabolic syndrome. J Obes Overweight.

[10] Reuters T (2012). Web of Science.

[11] Pokharel Y, Basra S, Lincoln AE, Tucker AM, Nambi V (2014). Association of body mass index and waist circumference with subclinical atherosclerosis in retired NFL players. South Med J.

[12] Abernethy WB, Choo JK, Hutter AM (2003). Echocardiographic characteristics of professional football players. J Am Coll Cardiol.

[13] George CF, Kab V, Kab P, Villa JJ, Levy AM (2003). Sleep and breathing in professional football players. Sleep Med.

[14] Ripka WL, Modesto JD, Ulbricht L, Gewehr PM (2016). Obesity impact evaluated from fat percentage in bone mineral density of male adolescents. PLoS One.

[15] Halson SL (2014). Sleep in elite athletes and nutritional interventions to enhance sleep. Sports Med.

[16] Batool AS, Goodwin JL, Kushida CA, Walsh JA, Simon RD (2016). Impact of continuous positive airway pressure (CPAP) on quality of life in patients with obstructive sleep apnea (OSA). J Sleep Res.

